# Seroprevalence and Associated Risk Factors of Foot and Mouth Disease in Cattle in West Shewa Zone, Ethiopia

**DOI:** 10.1155/2020/6821809

**Published:** 2020-03-31

**Authors:** Beyan Ahmed, Lencho Megersa, Getachew Mulatu, Mohammed Siraj, Gelma Boneya

**Affiliations:** ^1^Department of Veterinary Science, College of Agriculture and Veterinary Science, Ambo University, P.O. Box 19, Ambo, Ethiopia; ^2^Department of Veterinary Laboratory Technology, College of Agriculture and Veterinary Sciences, Ambo University, P.O. Box 19, Ambo, Ethiopia

## Abstract

Foot and mouth disease (FMD) is a highly contagious viral disease of cloven-hoofed animals and one of the endemic diseases in Ethiopia. The study was aimed to estimate the seroprevalence and to assess associated risk factors of foot and mouth disease seroprevalence in West Shewa Zone. A total of 384 sera samples were collected from randomly selected cattle and tested using ELISA for antibodies against nonstructural proteins of foot and mouth disease viruses based on IDEXX FMD Multispecies Ab Test (IDEXX Laboratories Inc, USA). The seroprevalence of foot and mouth disease in West Shewa Zone was found to be 40.4% (95% CI: 35.46–45.27) at an animal and 74.7% (95% CI: 65.58–83.85) at the herd level. Multivariable logistic regression analysis indicated that districts, breed, and animal composition were the potential risk factors of FMD seropositivity. Accordingly, cattle found in Abuna Ginde Beret (odds ratio (OR): 9.1, 95% CI: 2.4–34.1, *p*=0.001), Cheliya (OR: 8.8, 95% CI: 2.5–31.3, *p*=0.001), Bako Tibe (OR: 7.6, 95% CI: 2.1–28.3, *p*=0.002), Tokekutaye (OR: 5.8, 95% CI: 1.7–19.5, *p*=0.004), and Jeldu (OR: 5.3, 95% CI: 1.3–21.5, *p*=0.020) districts were more at risk to be infected with FMD than cattle from Ambo. The odds of FMD seropositivity was significantly higher in cattle kept with small ruminants (OR: 2.1, 95% CI: 1.3–3.3, *p*=0.003) than cattle alone. The analysis also revealed that the odds of seropositivity were 6 times higher in crossbred compared with local cattle (*p*=0.003). The current study found high seroprevalence of FMD in West Shewa Zone. Therefore, cattle should be vaccinated regularly after the identification of specific FMD serotypes circulating in the study area.

## 1. Introduction

Foot and mouth disease (FMD) is a severe highly contagious viral disease caused by an *Aphthovirus* of the family *Picornaviridae*. FMD virus has seven serotypes: A, O, C, Asia 1, SAT (Southern African Territories) 1, 2, and 3 [1]. Serotypes reported in Ethiopia are O, A, C, SAT1, and SAT2 [2]. There is also diversity of strains within each serotype [3]. The disease is characterized by fever, loss of appetite, salivation, and vesicular eruptions in the mouth, on the feet, and teats [1]. FMD primarily affects cloven-hoofed animals including cattle, pigs, sheep, goats, and experimental infections in alpacas and llamas [4]. The morbidity rate of the disease reaches 100% in cloven-hoofed animals with high mortalities in young animals [1, 5]. FMD was once distributed worldwide but has been eradicated in some regions, including North America and Western Europe [6].

The occurrence of the disease leads to loss of production, restriction of exports, and other socio-economic problems in the area. The direct impact of FMD includes meat and milk production losses, loss of drought power, lower weight gains, fertility problems, changes in herd structure, delay sale of cattle and products, and death of cattle, while the indirect impacts include additional cost of treatment, vaccination, vaccine delivery, movement control, diagnostic tests, culled cattle, and denied access to both local and international markets [7].

FMD currently is widely prevalent and distributed in all areas of Ethiopia, although the level of the disease prevalence may show significant variations across the different farming systems and agroecological zones of the country [8, 9]. Previously, the disease used to frequently occur in the pastoral herds of the marginal low-land areas of the country. However, this trend has changed, and the disease is frequently noted in the central/highland parts of the country [8, 9]. The seroprevalence investigations undertaken so far in the different parts of the country reported the prevalence that ranges from 5.6% to 53.6% in cattle [10–22]. In the current study area, knowing the status of the disease is very important because of a high cattle population and cattle marketing activities, the practice of communal grazing and watering and cattle movement. Although several studies have been conducted on the seroprevalence and associated risk factors of FMD in cattle in different parts of the country, there is a scarcity of information in the study area. Thus, implementing both seroprevalence and associated risk factors investigation is crucial to generate baseline information about the disease. Therefore, the objectives of the present study were to estimate the seroprevalence and to assess associated risk factors of FMD seroprevalence in West Shewa Zone.

## 2. Material and Methods

### 2.1. Study Area and Animals

The study was conducted in Jeldu, Cheliya, Bako Tibe, Abuna Ginde Beret, Ambo, and Tokekutaye districts of West Shewa Zone, Ethiopia, from December 2017 to September 2019. West Shewa Zone is one of the zones in the centre of the Oromia Regional State, Ethiopia. The zone is divided into 18 districts and 1 urban local administration. West Shewa Zone borders to Southwest Shewa and the Southern Nations, Nationalities and Peoples Region to the south, Jimma to the southwest, East Wollega to the west, Horo Guduru Wollega to the northwest, the Amhara Region to the north, North Shewa to the northeast, and Oromia Special Zone surrounding Addis Ababa to the east.

The altitude of the zone ranges from 1166–3238 meters above sea level (masl), and most areas lie between 2300 and 2630 masl. The topography of the zone is flat which makes the area an ideal place for agriculture. The mean annual temperature and rainfall range from 11–21°C and 880–1200 mm, respectively. The crop-livestock mixed farming system is a common practice. This zone is an ideal place for market-oriented commodity development as it is endowed with resources necessary for production and has good access to urban markets such as Addis Ababa, Holeta, and Ambo towns. This zone has a human population of 2,058,676, of which 1,028,501 are males and 1,030,175 are females [23]. Cattle population in the zone was estimated at 2.0 million, while sheep and goat population was 841,001 and 392,473, respectively [24].

The study animals were cattle that are kept under different management systems (extensive, intensive, and semi-intensive farming systems). All local and crossbred cattle of 2 years old and above had a chance to be included in the study.

### 2.2. Study Design and Sampling Techniques

The cross-sectional study design was employed to assess the seroprevalence of foot and mouth disease and to determine associated risk factors of FMD seroprevalence. Six districts of West Shewa Zone were included in the study based on agroecological conditions. The sample size was calculated based on the following formula [25]:

(1) $$\begin{array}{c} N=\frac{1.96^{2}\;P_{\exp}\left(1-P_{\exp}\right)}{d^{2}}, \end{array}$$

where *N* = sample size, *P*_exp_ = expected prevalence, and *d* = absolute precision.

5% absolute precision and 95% confidence interval were used to determine the sample size. Accordingly, the minimum sample size calculated was 384 samples.

Six districts were selected by primarily stratifying the districts based on their similarities in agroecological conditions, and then a simple random sampling method was used to select two districts from each stratum. In each district, Kebeles, households, and study animals were selected randomly.

### 2.3. Sample Collection

The blood was collected from the jugular vein of individual animals using a 10 ml plain vacutainer tubes and then allowed to clot to room temperature overnight. The sera were harvested on a separate tube and labeled. The collected sera were transferred into sterile cryovials and transported in an icebox to the Veterinary Microbiology Laboratory of Ambo University and stored at −20°C. Finally, at the end of each sampling, the sera were transported under the cold chain to the National Veterinary Institute (NVI), Bishoftu, and stored at −20°C [1].

### 2.4. Serological Diagnosis

#### 2.4.1. Serological diagnostic tests

The IDEXX FMD Multispecies Ab ELISA Test (IDEXX Laboratories, Inc, USA) was conducted to detect antibodies against nonstructural proteins (NSP) of the FMD virus in serum. IDEXX FMD Multispecies Ab Test provides a rapid, simple, sensitive, and specific method for detecting antibodies against nonstructural proteins (NSP) of the FMD virus in serum bovine origin. This test allows differentiation between samples from infected (presence of antibodies against NSP of FMD virus) and vaccinated (no antibodies against NSP of FMD virus) animals. The test was performed as per the manufacturer's instructions (IDEXX Laboratories Inc). The optical density (OD) reading was recorded using a spectrophotometer at a wavelength of 450 nm [26].

#### 2.4.2. Validity Criteria

The test result was validated if

- The mean value of the negative control (NC) is less than or equal to 0.3 (NCx̄ ≤ 0.3)
- The mean value of the positive control (PC) is less than or equal to 2 (PCx̄ ≤ 2)
- PCx̄-NCx̄ ≥ 0.3

#### 2.4.3. Interpretation

For each serum sample, the competition percentage was calculated (S/P%) as follows:

(2) $$\begin{array}{c} \left(\frac{S}{P}\right)\%=100\;\times \;\frac{\text{sample }A\;\left(450\right)-\text{NC}\bar{x}}{\text{PC}\bar{x}-\text{ NC}\bar{x}}. \end{array}$$

S/P% less than 35 was recorded as negative, and S/P% greater than or equal to 35 was positive.

### 2.5. Data Management and Analysis

Raw data were filled and coded in Microsoft Excel. Descriptive statistical analysis and univariable and multivariable logistic regressions were carried out using STATA software version 14.2 [27]. Odds ratio (OR) was calculated for each risk factor for seropositivity to FMD. In all the analyses, confidence levels at 95% were calculated, and *p* < 0.05 was used for the statistical significance level. OR was calculated to determine the degree of association between risk factors and seropositivity to the virus.

## 3. Results

### 3.1. Seroprevalence

In the present study, a total of 384 cattle of 87 herds were examined for the presence of antibodies against nonstructural proteins (NSP) of FMD virus in their blood sample using IDEXX FMD Multispecies Ab Test. Out of the total sera collected, the seroprevalence of FMD recorded at an animal and herd level was 40.4% (95% CI: 35.46–45.27) and 74.7% (95% CI: 65.58–83.85), respectively.

### 3.2. Association with Risk Factors

Table 1 shows the association of risk factors with animal FMD seropositivity. Accordingly, seropositivity significantly varied (*p* < 0.05) with districts, breed, management, and presence of small ruminants in the cattle herd.

## 4. Discussions

The current seroprevalence of FMD was 40.4%, which agreed with the seropositivity of 41.5% found in the eastern part of Tigray [14]. Besides, the seroprevalence of FMD in the present study was in agreement with the finding of Tekleghiorghis [28] who reported 39% seroprevalence in neighboring Eritrea.

Compared to the current findings, lower seroprevalence was reported from western Ethiopia (9%), Dire Dawa (8.1%), southern Ethiopia (9.5%), South Omo Zone (8.9%), East Shewa Zone (10.9%), and Amhara region (14.4%) [15, 20, 29–32]. On the contrary, higher seroprevalence of FMD was reported by Mekonnen et al. [11], Kibore et al. [33], and Lazarus et al. [34] from Ethiopia, Kenya, and Nigeria with the prevalence of 53.6%, 52.5%, and 72.6%, respectively. FMD is endemic in Ethiopia, and its prevalence ranges from 5.6% to 42.7% in different parts of the country [8]. Those differences of figures of prevalence reported by different scholars might be due to variation in a management system, free movement of cloven-hoofed animals, unequal distribution of vaccine through the country, intervention and agroclimatic condition, and presence of communal grazing and watering in the current study area.

The 74.7% seropositivity at the herd level in this study was higher than the reports of Megersa et al. [31] in southern Ethiopia and Hussain et al.'s study [35] in Oman with seroprevalence of 48.1% and 55.2%, respectively. The higher prevalence of FMD at the herd level might be due to the common practice of communal grazing and watering in the study area.

The present study showed that there was a statistically significant difference among districts. This is in line with the report of Beyene et al. [32] in western Ethiopia. The prevalence of FMD was higher in Abuna Ginde Beret and Cheliya than in Ambo. This might be due to differences in the unrestricted cattle movement [31]. Abuna Ginde Beret and Cheliya districts practice more communal grazing and watering compared to Ambo.

In the current study, cattle kept with small ruminants had higher odds (2.1 times) of infection than those kept alone (*p* < 0.5). This report corresponds with the studies of Sulayeman et al. [22], Beyene et al. [32], and Gelaye et al. [36]. Earlier studies confirmed that sheep and goats are important reservoirs of FMD virus infection in cattle [36].

In this study, the prevalence of FMD seropositivity was higher in females than males. In similar observation, Mazengia et al. [37], Mesfine et al. [30], and Mekonnen et al. [38] reported that the seroprevalence of bovine FMD was higher in females than males.

In the present study, significantly higher FMD seroprevalence was recorded in crossbred than local cattle. Similarly, higher FMD seroprevalence in crossbred than local cattle was reported by Sulayeman et al. [22] in Ethiopia. The relative higher seroprevalence in crossbred cattle might be attributed to the genetic variation among the breed of animals [1, 9, 39].

## 5. Conclusions

In conclusion, the findings of the present study revealed that foot and mouth disease is highly prevalent in West Shewa Zone. Statistical analysis indicated that districts, breed, and animal composition in cattle herds are major predictors of the foot and mouth disease occurrence. Identification of specific FMD serotypes circulating in the study area and regular vaccination of cattle are encouraged.

## Figures and Tables

**Table 1:** Univariable logistic regression analysis of potential risk factors associated with the seropositivity of bovine FMD in West Shewa Zone.

Risk factors	Categories	No. of cattle sampled	No. of seropositive	Seroprevalence (%)	Odds ratio	95% CI	*p* value
Districts	Ambo	66	19	28.8	1	—	
	Bako Tibe	78	30	38.5	1.5	0.8–3.2	0.223
	Cheliya	50	28	56.0	3.2	1.5–6.8	0.004
	Abuna Ginde Beret	51	24	47.1	2.2	1.0–4.7	0.044
	Jeldu	43	13	30.2	1.1	0−2.5	0.871
	Tokekutaye	96	41	42.7	1.3	0.8–3.7	0.073
Sex	Male	175	64	36.6	1	—	
	Female	209	91	43.5	1.3	0.9–2.1	0.166
Breed	Local	248	89	35.9	1	-	
	Cross	136	66	48.5	1.7	1.1–2.6	0.016
Management	Extensive	316	117	37.0	1	—	
	Semi-intensive and intensive	68	38	55.9	2.2	1.3–3.7	0.005
Sheep and goats in cattle herd	No	152	42	27.6	1	—	
	Yes	232	113	48.7	2.4	1.6–3.9	≤0.001
Age in years	2–4	185	72	38.9	1	—	
	>4	199	83	41.7	1.1	0.8–1.7	0.578
Common grazing	No	159	59	37.1	1	—	
	Yes	225	96	42.7	1.3	0.8–1.9	0.274

The multivariable logistic regression model of risk factors analysis indicated that districts, breed, and animal composition in herds had a significant association with seroprevalence of FMD and hence are independent predictors (*p* < 0.05). Abuna Ginde Beret and Cheliya districts have a chance of 9 times more likely to have FMD seropositive when compared to the Ambo district. The prevalence of FMD was higher when cattle keeping with small ruminants, and the data had a statistically significant difference (OR: 2.1) (Table 2).

**Table 2:** Results of the multivariable logistic regression model for the predictors of seropositivity of bovine FMD in the West Shewa Zone.

Risk factors	Categories	Odds ratio	95% CI	*p* value
Districts	Ambo	1	—	
	Jeldu	5.3	1.3–21.5	0.020
	Tokekutaye	5.8	1.7–19.5	0.004
	Bako Tibe	7.6	2.1–28.3	0.002
	Cheliya	8.8	2.5–31.3	0.001
	Abuna Ginde Beret	9.1	2.4–34.1	0.001
Breed	Local	1	—	
	Cross	6.30	1.9–20.9	0.003
Sheep and goats in cattle herd	No	1	-	
	Yes	2.1	1.3–3.3	0.003
Management	Extensive	1	—	
	Semi-intensive and intensive	0.46	0.1–1.5	0.197
Sex	Male	1	—	
	Female	1.0	0.6–1.6	0.982

## Data Availability

The data used to support the findings of this study are available from the corresponding author upon request.

## Abbreviations

FMD: Foot and mouth disease
FMDV: Foot and mouth disease virus
CI: Confidence interval
OR: Odds ratio
CSA: Central statistical agency
NSP: Nonstructural proteins
OD: Optical density
Ab: Antibody
